# Impact of the COVID-19 pandemic on congenital diaphragmatic hernia patients: a single-center retrospective study

**DOI:** 10.1007/s00383-022-05136-9

**Published:** 2022-06-07

**Authors:** Yun-Long Zhao, Ying Wang, Chao Liu, Yu-Lin Jiang, Yan-Dong Wei, Hua Meng, Shan Jian, Xi-Ting Zhu, Li-Jian Pei, Xiao-Chen Bai, Feng Feng, Yan Lv, Xi-Ya Zhou, Qing-Wei Qi, Jing-Na Li, Wei Ji, Li-Shuang Ma

**Affiliations:** 1 Department of Neonatal Surgery, Children’s Hospital of Capital Institute of Pediatrics, Graduate School of Peking Union Medical College, Beijing, China; 2 Department of Neonatal Surgery, Children’s Hospital of Capital Institute of Pediatrics, Beijing, China; 3 Department of Obstetrics, Peking Union Medical College Hospital, Beijing, China; 4 Department of Ultrasound, Peking Union Medical College Hospital, Beijing, China; 5 Department of Pediatrics, Peking Union Medical College Hospital, Beijing, China; 6 Everest Clinical Research Corporation, New Jersey, USA; 7 Department of Anesthesiology, Peking Union Medical College Hospital, Beijing, China; 8 Department of Pediatrics, Beijing Obstetrics and Gynecology Hospital, Capital Medical University, Beijing, China; 9 Department of Radiology, Peking Union Medical College Hospital, Beijing, China; 10 Department of Interventional Hemangioma, Children’s Hospital of Capital Institute of Pediatrics, Beijing, China

**Keywords:** Public health emergency, Neonates, Congenital diaphragmatic hernia, Surgery, Clinical management

## Abstract

**Purpose:**

To investigate the impact of COVID-19 on the treatment of children with congenital diaphragmatic hernia (CDH).

**Methods:**

We retrospectively collected and compared the data of patients with CDH admitted between January 1, 2020 and December 31, 2021(study group) with the CDH patients admitted before the pandemic between January 1, 2018 and December 31, 2019 (control group).

**Results:**

During the pandemic, 41 patients with CDH diagnosed prenatally were transferred to our hospital, and 40 underwent surgical repair. The number of patients treated in our hospital increased by 24.2% compared with the 33 patients before the pandemic. During the pandemic, the overall survival rate, postoperative survival rate and recurrence rate were 85.4%, 87.5% and 7.3%, respectively, and there were no significant differences compared with the control group (75.8%, 83.3% and 9.1%, respectively). The average length of hospital stay in patients admitted during the pandemic was longer than that in the control group (31 days vs. 16 days, *P* < 0.001), and the incidence of nosocomial infection was higher than that in the control group (19.5% vs. 3%, *P* = 0.037).

**Conclusions:**

CDH patients confirmed to be SARS-CoV-2 infection-free can receive routine treatment. Our data indicate that the implementation of protective measures during the COVID-19 pandemic, along with appropriate screening and case evaluation, do not have a negative impact on the prognosis of children.

## Introduction

Congenital diaphragmatic hernia (CDH) is caused by fetal congenital diaphragmatic hypoplasia and abdominal organs herniating into the thoracic cavity, leading to neonatal pulmonary hypoplasia. CDH occurs in approximately 2.6 of 10,000 live births [1]. Due to the abdominal organs that herniate into the chest cavity during the development process, the lungs are squeezed and hypoplastic. Children often develop pulmonary hypertension, cardiopulmonary insufficiency and respiratory failure immediately after birth. Therefore, a prompt surgical treatment at birth is mandatory. Although surgical techniques and neonatal intensive care have undergone significant progress in the past few decades, the mortality rate of children with severe CDH is still high, reaching 70% [2].

**Fig. 1:**
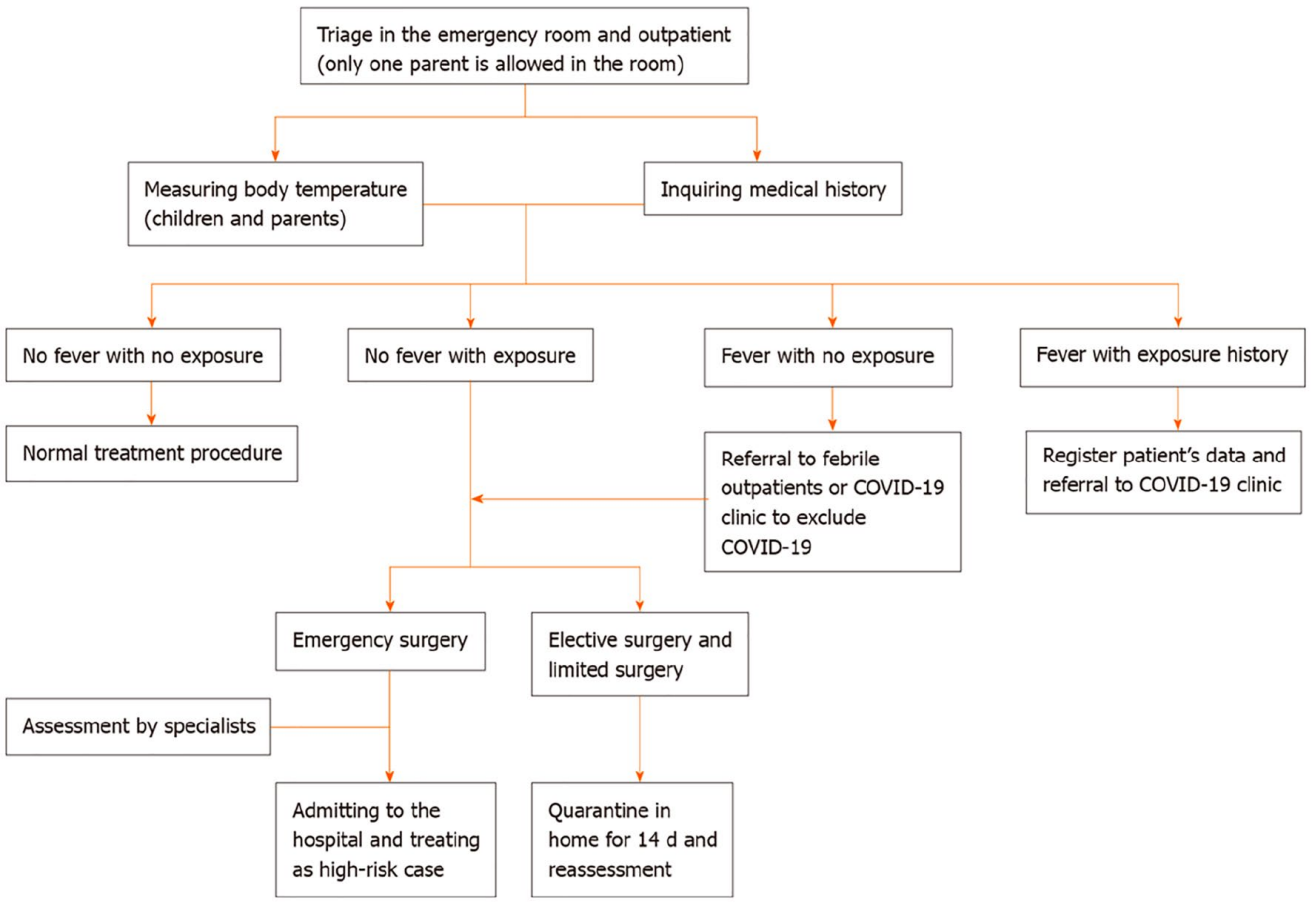
Diagnosis and treatment strategies for the neonatal outpatient service and emergency room during the coronavirus disease 2019 pandemic. COVID-19: Coronavirus disease 2019. Ma LS, Zhao YL, Wei YD, Liu C. Recommendations for perinatal and neonatal surgical management during the COVID-19 pandemic. World Journal of Clinical Cases. 2020;8(14):2893–901. This figure is from a previous publication by our team

**Fig. 2:**
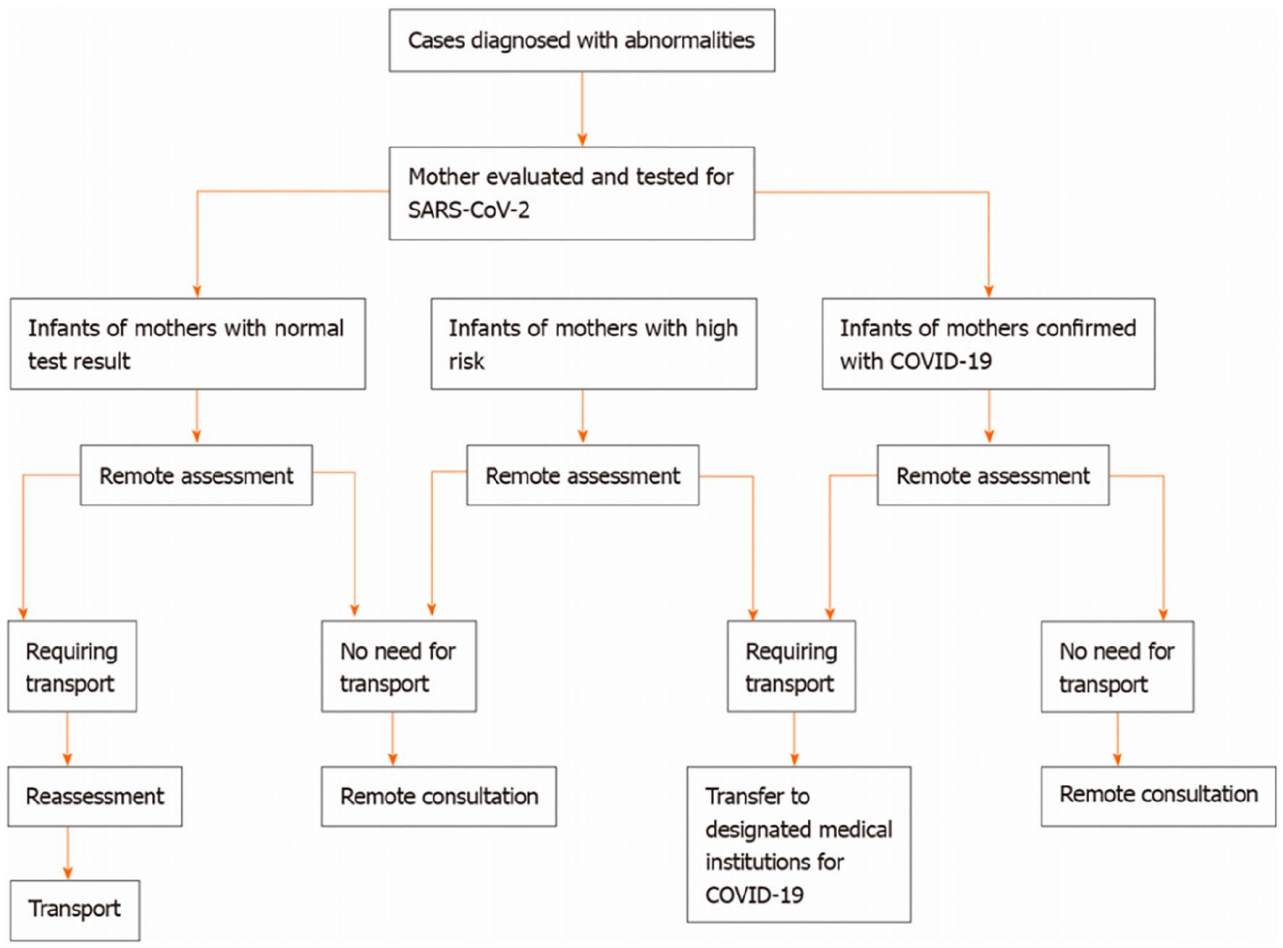
Transportation strategies for severe and critically ill patients from the neonatal surgery department during the coronavirus disease 2019 pandemic. COVID-19: Coronavirus disease 2019; SARS-CoV-2: Severe acute respiratory syndrome coronavirus 2. Ma LS, Zhao YL, Wei YD, Liu C. Recommendations for perinatal and neonatal surgical management during the COVID-19 pandemic. World Journal of Clinical Cases. 2020;8(14):2893–901. This figure is from a previous publication by our team

**Fig. 3:**
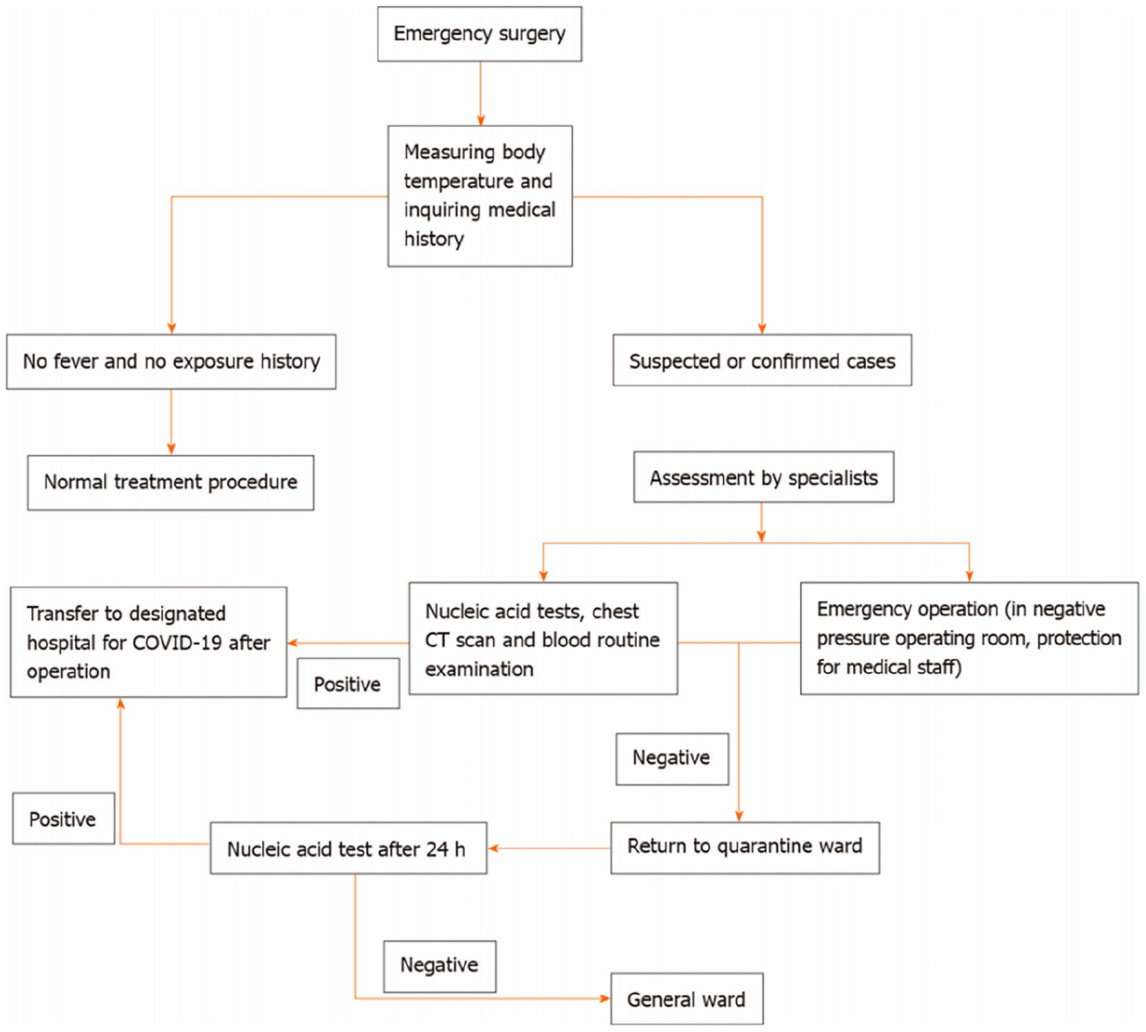
Neonatal emergency surgery procedures in the neonatal surgery department during the coronavirus disease 2019 pandemic. COVID-19: Coronavirus disease 2019; CT: Computed tomography. Ma LS, Zhao YL, Wei YD, Liu C. Recommendations for perinatal and neonatal surgical management during the COVID-19 pandemic. World Journal of Clinical Cases. 2020;8(14):2893–901. This figure is from a previous article by our team

Since December 2019, severe acute respiratory syndrome coronavirus 2 (SARS-CoV-2), the etiologic agent of coronavirus disease 2019 (COVID-19), has spread rapidly, posing a significant threat to global health. The COVID-19 pandemic poses major challenges to antenatal care, delivery, as well as patient transport, and surgery of children with abnormal prenatal screening, requiring major adjustments to the global health care system. Therefore, knowing the impact of COVID-19 on the mortality rate and recurrence rate of children with abnormal prenatal screening is critical; thus, improving the management of neonatal patients during the pandemic is a top priority. As a tertiary referral hospital and one of the National Clinical Research Centers for Child Health and the National Children’s Medical Centers, our center provides care for a large proportion of prenatal and neonatal cases in north China. With the aim of improving transportation and surgery strategies for patients and preventing infection in medical staff during the current COVID-19 pandemic, we have developed recommendations focusing on practices, such as patient transport, surgery selection and protection requirements in the neonatal surgery department[3] (Figs. 1, 2, 3). However, the effect of these procedures on children with congenital structural malformations is unclear, and relevant research is lacking.

The present retrospective study compared the clinical data of children with CDH treated at the Children’s Hospital of Capital Institute of Pediatrics from January 1, 2020 to December 31, 2021 during the period of the COVID-19 pandemic with those treated from January 1, 2018 to December 31, 2019 (before the pandemic). The purpose of this study was to report our experience in implementing these prevention procedures and treating CDH during the COVID-19 pandemic, to evaluate the impact of the new prevention procedures and related protective measures on the prognosis of children with prenatally diagnosed CDH, and to share the experience and lessons learned by us during this public health emergency.

## Materials and methods

### Study design and setting

**Fig. 4:**
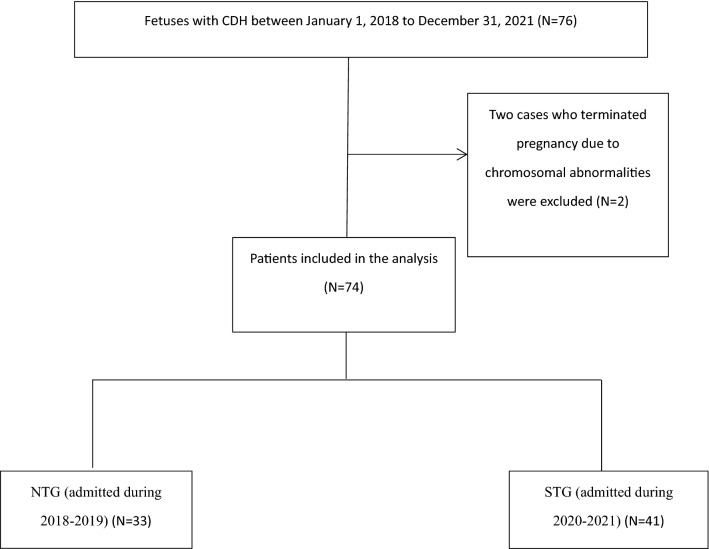
Study flow diagram. CDH congenital diaphragmatic hernia; NTG Normal time group; STG Special time group

This matched study at the Children’s Hospital of Capital Institute of Pediatrics (Beijing) was approved by the hospital Ethics Committee (SHERLLM2022009). Inclusion criteria were: (1) definite prenatal diagnosis of CDH from January 2018 to December 2021 (diagnosis and evaluation were carried out by ultrasound and/or MRI); (2) treated at our hospital after birth; and (3) complete medical records. Exclusion criteria were: (1) not being treated for the first time in our hospital after birth; (2) incomplete medical records; (3) chromosomal abnormalities; (4) miscarriage or termination of pregnancy; and (5) non-CDH death (Fig. 4).

### Interventions

Since January 2020, our hospital has carried out medical activities in accordance with the “Guideline for the Diagnosis and Treatment of COVID-19 Infections” (version 7) (National Health Commission of China) [4], and formulated corresponding management strategies according to the characteristics of newborns [3]. For the children transferred to our center, according to the guidelines of the Canadian CDH Collaborative [5], if the children met the surgical indications, surgery to repair the diaphragm defect was performed. In this study, the children were divided into the normal-time group (NTG) and special-time group (STG) according to the time of admission in 2018–2019 (before the COVID-19 pandemic) and 2020–2021 (during the COVID-19 pandemic).

### Data collection

We included survival at discharge as the main outcome. The patients’ information included: gestational age at birth; gestational age at diagnosis (gestational age at which CDH was found on the first ultrasound screening or MRI examination); gender; birth weight; heart structural abnormalities; chromosomal abnormalities; presence of liver hernia; and whether a patch was used during the operation. We also recorded the affected side (left/right) and the classification (A–D) based on the size of the diaphragmatic defect using Lally’s criteria [6]. We used the postpartum risk prediction model developed by Brindle [7] to divide patients into low, medium and high risk based on scores of 0, 1–2, and ≥ 3, respectively.

### Statistical analysis

Continuous data were expresses as Mean (IQR) and dichotomous data were expresses as *n*(%). Data processing was conducted using SPSS version 25.0. Continuous data were compared between the NTG and STG using the *t* test for normally distributed variables and the Mann- Whitney *U* test for non-parametric variables. Dichotomous variables were assessed with the χ2 test or Fisher’s exact test. A *P* value < 0.05 was considered statistically significant.

## Results

From 2018 to 2021, a total of 76 cases of prenatally diagnosed CDH were identified in our multidisciplinary clinic, of which 2 cases terminated the pregnancy due to chromosomal abnormalities, and 74 cases were transferred to our center after delivery. Thirty-three patients were born and treated in 2018–2019 (NTG) and 41 patients were born and treated in 2020–2021 (during the COVID-19 pandemic, STG). Table 1 summarizes the clinical data of the 74 patients included in the study.

**Table 1:** Characteristics of patients who received treatment before and during the pandemic

Parameters	Total *N* = 74	NTG^a^ (2018–2019) *N* = 33	STG^b^ (2020–2021) *N* = 41	*P* value
EXIT^c^	35 (47.3%)	6 (18.2%)	29 (70.7%)	< 0.001*
Survival of EXIT	82.9% (29/35)	83.3% (5/6)	82.8% (24/29)	1
Gender				0.093
Female	28 (37.8%)	9 (27.3%)	19 (46.3%)	
Male	46 (62.2%)	24 (72.7%)	22 (53.7%)	
Method of delivery				0.176
Cesarean section	65 (87.8%)	27 (81.8%)	38 (92.7%)	
Vaginal delivery	9 (12.2%)	6 (18.2%)	3 (7.3%)	
Site				0.519
Left	58 (78.4%)	27 (81.8%)	31 (75.6%)	
Right	16 (21.6%)	6 (18.2%)	10 (24.4%)	
Surgical repair	70 (94.6%)	30 (90.9%)	40 (97.6%)	0.318
Surgery approach *N* = 70				0.218
MIS	54 (77.1%)	21 (70%)	33 (82.5%)	
Other	16 (22.9%)	9 (30%)	7 (17.5%)	
Defect Size *N* = 67				0.711
A	0	0	0	
B	7 (10.4%)	3 (11.1%)	4 (10%)	
C	45 (67.2%)	17 (63%)	28 (70%)	
D	15 (22.4%)	7 (25.9%)	8 (20%)	
Hernial sac *N* = 70	18 (25.7%)	8 (26.7%)	10 (25%)	0.875
Patch *N* = 70	12 (17.1%)	5 (16.7%)	7 (17.5%)	0.927
Any portion of liver in chest *N* = 73	20 (27.4%)	9 (27.3%)	11 (27.5%)	0.983
Survival	60 (81.1%)	25 (75.8%)	35 (85.4%)	0.294
Survival after operation *N* = 70	60 (85.7%)	25 (83.3%)	35 (87.5%)	0.735
Survival after MIS	50 (92.6%)	19 (90.5%)	31 (93.9%)	0.638
Other structural abnormalities	8 (10.8%)	5 (15.2%)	3 (7.3%)	0.454
Recurrence after operation *N* = 70	6 (8.1%)	3 (9.1%)	3 (7.3%)	1
Recurrence after MIS	4 (7.4%)	2 (9.5%)	2 (6.1%)	0.638
Nosocomial infection	9 (12.2%)	1 (3%)	8 (19.5%)	0.037*
Pulmonary hypertension				0.001*
Normal	15 (20.3%)	13 (39.4%)	2 (4.9%)	
Mild	19 (25.7%)	9 (27.3%)	10 (24.4%)	
Moderate	17 (23%)	4 (12.1%)	13 (31.7%)	
Severe	23 (31%)	7 (21.2%)	16 (39%)	

Data are presented as *n*(%)

*A *p* value < 0.05 was considered statistically significant

^a^*NTG* normal time group

^b^*STG* special time group

^c^*EXIT* *ex utero* intrapartum treatment

All 41 children with CDH in STG were diagnosed prenatally, and their mothers' qRT-PCR test were negative before delivery. All the children were transferred from the maternity hospital to our hospital after birth according to the transportation strategies (Fig. 2). After admission, they were placed in a quarantine chamber care unit for treatment, and transferred to neonatal intensive care unit for further treatment after qRT-PCR test was confirmed negative. The intensive care unit was nonescort ward, and parent visiting was cancelled to avoid infection. None of the patients tested positive by qRT-PCR or blood SARS-CoV-2 antibodies by the time of discharge. In general, there were no statistically significant differences between the NTG and STG in terms of gestational age at birth, gestational age at diagnosis, birth weight, pH of the first blood gas analysis after admission, location and size of the diaphragm defect, surgical approach, operation time, hepatic hernia, presence of a hernia sac, pulmonary hypertension, and severe structural malformation (Tables 1 and 2).

**Table 2:** Clinical data of patients who received treatment before and during the pandemic

Parameters	NTG^a^ (2018–2019) *N* = 33	STG^b^ (2020–2021) *N* = 41	*P* value
PH(First blood gas after admission)	7.26 [7.17–7.37]	7.33 [7.285–7.4]	0.058
Mechanical ventilation time (d)	7 [1–11]	15 [6–24]	0.002*
Length of stay (d)	16 [9–23]	31 [20–39.5]	< 0.001*
Age at admission (h)	2 [1–4]	1 [1–1]	< 0.001*
Gestational age at diagnosis (w)	28 [23–32]	24 [23.5–30]	0.221
Gestational age at birth (w)	37.5 [37–38.05]	37.3 [37.1–37.6]	0.563
Birth weight(kg)	3 [2.7–3.435]	3 [2.65–3.24]	0.687
Age at operation *N* = 70(h)	25.5 [24–29.25]	27 [25–43]	0.087
Operation time *N* = 69 (min)	180 [130.5–220]	150 [118.5–193]	0.124
Apgar 1 *N* = 45	9 [8–10]	9 [8, 9]	0.829
Apgar 5 *N* = 44	9 [9, 10]	9 [9, 10]	0.562
Brindle	2 [1, 2]	2 [1, 2]	0.902
Cost (¥10,000)	9.73 [7.07–13.79]	17.41 [10.56–24.46]	< 0.001*

Data are expresses as mean (IQR)

*A *p* value < 0.05 was considered statistically significant

^a^*NTG* normal time group

^b^*STG* special time group

Since 2019, for some children prenatally diagnosed with CDH, together with the maternity hospital, we have adopted a method of fetal resuscitation by endotracheal intubation and effective mechanical ventilation followed by clamping the umbilical cord through EXIT (ex utero intrapartum treatment) during labor. Of 33 patients in the NTG, 6 patients received EXIT, and 5 of them survived, with a survival rate of 83.3%. Of the 41 patients in the STG, 29 received EXIT, of which 24 survived, with a survival rate of 82.8%.

In NTG, 30/33 (90.9%) were treated with surgery, and 3 (9.1%) died before surgery due to severe pulmonary hypertension, including one CDH patient with left ventricular dysplasia. In STG, 40/41 (97.6%) were treated with surgery and 1 (2.4%) patient who died before surgery due to severe pulmonary hypertension. The overall survival rates in the two groups were 75.8% and 85.4%, respectively, and the postoperative survival rates were 83.3% and 87.5%, respectively, with no statistically significant differences. The postoperative recurrence rates in the two groups were 9.1% and 7.3%, respectively, with no statistically significant difference. The postoperative survival rate in the children who underwent thoracoscopic surgical repair before and during the pandemic was 90.5% and 93.9%, respectively, and the postoperative recurrence rate in the children who underwent thoracoscopic surgical repair was 9.5% and 6.1%, respectively, with no statistically significant differences. Pulmonary hypertension in STG group was more serious than that in NTG group (*P* = 0.001).

The length of hospital stay of STG was 31[IQR, 20–39.5] days, which was longer than the length of hospital stay (16[IQR, 9–23] days) of the NTG (*P* < 0.001). The mechanical ventilation time of STG was 15[IQR, 6–24] days, which was longer than the mechanical ventilation time (7[IQR, 1–11] days) of the NTG (*P* = 0.002). The total hospital costs for patients in the STG were also higher than those in the NTG. The median total expenses of patients in the NTG and STG were ¥97,300 RMB and ¥174,100 RMB, respectively, and the difference was statistically significant (*P* < 0.001).

The number of nosocomial infections in the two groups was 1 (3%) and 8 (19.5%), respectively, showing a significant difference in the incidence of nosocomial infection (*P* = 0.037). These nine cases developed fever after surgery and were diagnosed by specimen culture. Eight had a bloodstream infection (BSI) and one had a central line-associated BSI (CLABSI) (Table 3). Compared to those without nosocomial infection, the intubation time, length of stay and cost in the nosocomial infection group were greater than those in the nosocomial infection-free group. The outcomes of the 9 patients with nosocomial infection were 1 death and 1 postoperative recurrence, and all of the two were admitted during the pandemic period (STG). The survival and recurrence rate of patients with nosocomial infection were 88.9% and 11.1%, respectively, with no differences compared to those without nosocomial infection (80.0% and 7.7%, respectively; *P* > 0.05) (Table 4).

**Table 3:** Cases of BSI and CLABSI infection

Group	Serial number	Types of Infection	Cost(¥10,000)	PH(First blood gas after admission)	Mechanical ventilation time(d)	Length of stay(d)	Gestational age at diagnosis(w)	Gestational age at birth(w)	Birth weight(kg)	Survival	pulmonary hypertension	Brindle	Recurrence
NTG^a^	1	BSI^c^	16.7	7.09	17	22	36	38	3.6	Survival	Severe	2	NO
STG^b^	2	BSI	13.2	7.35	10	24	24	37.5	3.11	Survival	Mild-to-moderate	1	NO
STG	3	BSI	16.5	7.33	16	25	30	38.6	3.11	Survival	Mild-to-moderate	2	NO
STG	4	CLABS^d^	18.8	7.39	14	38	23	37.6	3.51	Survival	NO	2	NO
STG	5	BSI	28.7	7.31	0	43	23	38	3.6	Death	Mild-to-moderate	1	NO
STG	6	BSI	30.9	7.32	53	63	32	37.2	3	Survival	Moderate-severe	2	NO
STG	7	BSI	27.6	7.27	31	42	30	37.6	2.96	Survival	Severe	2	YES
STG	8	BSI	33.0	7.22	47	57	24	37.1	2.5	Survival	Severe	2	NO
STG	9	BSI	21.8	7.3	24	33	22	38.1	3.02	Survival	Moderate	2	NO

^a^*NTG* normal time group

^b^*STG* special time group

^c^*BSI* Bloodstream infection

^d^*CLABSI* Central line-associated bloodstream infection

**Table 4:** Characteristics associated with infection in infants with CDH

Parameters	Non-nosocomial infection *N* = 65	Nosocomial infection *N* = 9	*p* value
EXIT^a^	30 (46.2%)	7 (77.8%)	0.152
Site			0.396
Left	52 (80%)	6 (66.7%)	
Right	13 (20%)	3 (33.3%)	
Surgical repair	61 (93.8%)	9 (100%)	1
Surgery approach *N* = 70			0.218
MIS *n*/(*N*%)	48 (78.7%)	6 (66.7%)	
Other *n*/(*N*%)	13 (21.3%)	3 (33.3%)	
Defect Size *N* = 67			0.534
A	0	0	
B	6 (10.3%)	1 (11.1%)	
C	40 (69%)	5 (55.6%)	
D	12 (20.7%)	3 (33.3%)	
Hernial sac *N* = 70	43 (70.5%)	9 (100%)	0.099
Patch *N* = 70	9 (14.8%)	3 (33.3%)	0.177
Any portion of liver in chest *N* = 73	16 (25%)	4 (44.4%)	0.246
Survival	52 (80%)	8 (88.9%)	1
Other structural abnormalities	8 (12.3%)	0 (0%)	0.584
Recurrence after operation *N* = 70	5 (7.7%)	1 (11.1%)	0.554
PH(First blood gas after admission)	7.32 [7.215–7.4]	7.31 [7.245–7.34]	0.591
Mechanical ventilation time(d)	9 [3–17]	17 [12–39]	0.024
Length of stay(d)	19 [15–31.5]	38 [24.5–50]	0.004
Age at admission(h)	1 [1, 2]	1 [1–1]	0.348
Gestational age at diagnosis(w)	26 [23.5–31]	24 [23–31]	0.927
Gestational age at birth(w)	37.3 [37–38]	37.6 [37.35–38.05]	0.119
Birth weight(kg)	3 [2.65–3.31]	3.11 [2.98–3.555]	0.17
Age at operation *N* = 70(h)	26 [24–32]	26 [24.5–48]	0.481
Operation time *N* = 69 (min)	167.5 [121.25–209]	120 [67–168.5]	0.053
Apgar 1 *N* = 45	9 [8–10]	9 [8.75–9.25]	0.71
Apgar 5 *N* = 44	9 [9, 10]	9 [9, 10]	0.851
Brindle	2 [1, 2]	2 [1.5–2]	0.456
Cost(¥10,000)	11.5 [7.7–17.8]	21.9 [16.6–29.8]	0.001

Dichotomous data are *n*(%), continuous data are mean(IQR)

^a^*EXIT* *ex utero* intrapartum treatment

As of this writing, no patient or medical staff in our center has been infected with SARS-COV-2 during the pandemic.

## Discussion

We previously advised on the perinatal and surgical management of newborns during the COVID-19 pandemic [3]. Not only for the prevention and control of COVID-19, but also to promote the orderly diagnosis and treatment of neonatal surgical diseases, including routine diagnosis and treatment, child transport and emergency surgical management (Figs. 1, 2 and 3). In the present study, we collected data from all CDH patients treated in our hospital during the COVID-19 outbreak and investigated the impact of the outbreak on the treatment of children with CDH.

Children with CDH often have pulmonary hypertension and respiratory failure after birth, with an overall mortality rate of 31–38% [1, 8, 9]; thus, immediate medical support and treatment after birth are essential for their survival. For CDH patients who met the surgical indications, our center continued to perform surgery as usual. An increase of number of patients during the pandemic (2020–2021) compared with that before the pandemic (2018–2019), indicating that we did not reduce our medical activities due to the pandemic. There was no significant difference in patient age at admission between the two groups before and after the pandemic, indicating that after the strict implementation of the transfer procedure, the time of transfer to our hospital did not increase, and the treatment of these children was not delayed. None of the children transferred to our center developed SARS-CoV-2 infection, suggesting that the process of transferring the children from the maternity hospital to the children’s hospital did not increase the risk of SARS-CoV-2 infection.

There was no statistically significant difference between the two groups (NTG and STG) of CDH children in terms of the lesion site (left and right sides), the size of the defect, liver herniation, the presence of a hernia sac and whether a patch was used during the operation, indicating that the pandemic and the related prevention and control measures did not reduce or delay the treatment of critically ill patients in our center. The postoperative recurrence rate of CDH is 3–26% [10, 11], and the recurrence rate after MIS (minimally invasive surgery) is higher than that of open surgery [12]. The postoperative recurrence rate and recurrence rate after thoracoscopic surgery of the STG were within the normal range. There were no significant differences in surgical approach, overall and postoperative survival rate and recurrence rate between the two groups. These data indicated that the implementation of pandemic prevention and control measures did not change the choice of surgical method for CDH in our center, and did not have an adverse impact on the prognosis of this population.

To improve the survival rate of children with CDH, since 2019, a method of fetal resuscitation by endotracheal intubation and mechanical ventilation before clamping the umbilical cord through EXIT has been adopted. The EXIT survival rates of the two groups showed no significant difference. No maternal complications, such as severe bleeding (blood loss ≥ 500 ml) and wound infection, or fetal complications related to EXIT occurred in all patients who received EXIT treatment. These results showed that the pandemic did not cause additional risks to puerpera and fetuses receiving this new treatment method.

Due to the need for surgical procedures and indwelling central lines, prolonged mechanical ventilation, and nutritional deficiencies, infants with CDH are at increased risk of nosocomial infection [13, 14]. A previously published study shows that a diagnosed BSI is one factor that predicts mortality or prolonged length of stay [15]. In this study, the incidence of nosocomial infection, length of stay and total medical expenses of hospitalized patients in the STG were significantly higher than those of patients in the NTG. Pulmonary hypertension in STG group was more serious than that in NTG group. Further analysis found that the total ventilation time, length of stay and cost of children with nosocomial infection were higher than those in the nosocomial infection-free group. It was shown that the occurrence of nosocomial infection is associated with prolonged hospital stay. These data are similar to existing research results. The prolonged hospital stay and additional costs for the treatment of infection led to an increase in the total cost. Our interpretation of this phenomenon is that, since pulmonary hypoplasia and pulmonary hypertension are two important factors affecting the survival rate of CDH patients [2], the more severe pulmonary hypertension in the STG group may have led to a longer ventilation time in the STG group, resulting in a longer hospital stay. In addition, the occurrence of nosocomial infection is associated with prolonged hospital stay. However, this explanation requires observation and analysis of more cases to confirm it.

Due to changes in medical expense pricing rules and medical insurance reimbursement policies in 2019–2020 in Beijing, the total hospitalization expenses for children admitted before and after that time changed accordingly. Based on our past experience, the actual cost burden of patients should be reduced as the proportion of national medical insurance reimbursement increases. However, the precise extent of the effect of insurance policy changes and infections is unclear, and more data are needed to clarify this issue.

This study was based on the analysis of real-world data from a single center, which may have led to some bias. Second, the results of our efforts during the pandemic have yet to be confirmed with longer follow-up times. In addition, the severity of the pandemic, public health resources, and cultural background vary from region to region, and some of our recommendations and methods may not be applicable to all regions.

In conclusion, our study showed that it is safe and feasible to treat children with CDH during COVID-19 when measures are put in place to reduce the risk of infection for patients and health care workers. Routine treatment and appropriate exploration of new therapies do not affect the outcome of children and do not increase the risk of SARS-CoV-2 infection for patients and medical staff. The incidence of nosocomial infection in children with prenatally diagnosed CDH admitted to hospital during the pandemic period is high, which deserves vigilance by clinicians. We hope that this study will be useful for physicians concerned with CDH.
